# Two-dimensional magnetic resonance imaging sequences correlate to three-dimensional computed tomography for evaluation of glenoid bone loss

**DOI:** 10.1016/j.jseint.2025.06.006

**Published:** 2025-07-02

**Authors:** Scott M. Feeley, Shankar S. Thiru, Benjamin E. Neubauer, Rachel E. Cherelstein, Christopher M. Kuenze, Udit Rawat, Edward S. Chang

**Affiliations:** a Walter Reed National Military Medical Center, Department of Orthopaedic Surgery, Bethesda, MD, USA; b Georgetown University School of Medicine, Washington, DC, USA; c Eastern Virginia Medical School, Norfolk, VA, USA; d Inova Sports Medicine, Falls Church, VA, USA; e Department of Kinesiology, University of Virginia, Charlottesville, VA, USA; f Fairfax Radiological Consultants, Fairfax, VA, USA; g Inova Health System, Falls Church, VA, USA; h University of Virginia School of Medicine, Fairfax, VA, USA

**Keywords:** Shoulder instability, GBL, MRI, CT, PD FS, FIESTA

## Abstract

**Background:**

To correlate two-dimensional (2D) fast-imaging employing steady-state acquisition (FIESTA) magnetic resonance imaging protocols with three-dimensional (3D) computed tomography (CT) for calculation of glenoid bone loss (GBL). FIESTA is a high-resolution protocol with high contrast, high signal-to-noise ratio, and sharp edge definition.

**Methods:**

We included patients who underwent a shoulder stabilization procedure from 2010 to 2023 with 2D FIESTA and 2D proton density fat-saturation (PD FS) magnetic resonance arthrogram sequences as well as 3D CT of the shoulder. We excluded patients with history of prior shoulder surgery or posterior instability, if the CT and magnetic resonance imaging were >90 days apart or if there was a documented instability event between imaging studies. Two raters calculated the primary outcome of GBL by the perfect-circle linear method twice for each modality separated by 3 weeks. Intrarater and inter-rater reliabilities were calculated. We analyzed linear regression models and concordance correlation coefficients between modalities.

**Results:**

Twelve patients were analyzed. Overall mean GBL by imaging modality was 12.1 ± 11.2% on PD FS, 11.8 ± 10.0% on FIESTA, and 11.5 ± 9.7% on 3D CT. GBL had excellent intrarater and inter-rater reliabilities (>0.9) on all modalities. For GBL, concordance correlation coefficients were 0.982 (95% CI 0.960-0.992) for PD FS vs. CT and 0.998 (95% CI 0.993-0.999) for FIESTA vs. CT. Linear regression models improved both PD FS and FIESTA accuracy to within 0.6% of CT-calculated GBL 95% of the time.

**Conclusion:**

2D FIESTA magnetic resonance arthrogram is a suitable alternative to 3D CT reconstruction for calculation of GBL and slightly outperformed PD FS sequences.

Anterior shoulder dislocation results in glenoid bone loss (GBL) of approximately 7% in first-time dislocators and recurrent instability frequently results in more than double GBL to greater than 20%.^1^^,^^7^ However, even smaller amounts of GBL of 13.5% have been associated with worse outcomes when unaddressed.^17^ The failure of soft-tissue stabilization procedures when treating patients with larger critical bone loss values has led to the use of bone augmentation for reconstruction of the native glenoid.^4^^,^^14^ Appropriate surgical management necessitates the ability to accurately diagnose GBL preoperatively.

Three-dimensional (3D) computed tomography (CT) is the gold standard imaging modality to measure GBL.^3^^,^^15^ The risk of failure associated with untreated critical GBL has led to recognition that precise measurement of GBL is needed in addition to examination of labral pathology on magnetic resonance imaging (MRI). This has amounted to a trend towards obtaining both MRI and CT preoperatively.^16^ More recently, there are MRI sequences that can be obtained with commercial MRI vendors and reconstructed three-dimensionally similar to 3D CT reconstructions.^10^ 3D MRI has been demonstrated as comparable to 3D CT both in vitro and in vivo.^23^^,^^25^ However, the processes for creating 3D reconstructions from MRI utilized in these studies are not widely available for clinical implementation. The ability to utilize routine two-dimensional (2D) MRI sequences to reliably measure GBL would obviate the need to obtain a preoperative CT prior to shoulder stabilization procedures, reducing time expended, medical resources used, and cost and radiation to the patient.^21^

Fast-imaging employing steady-state acquisition (FIESTA) MRI is a high-resolution protocol with high contrast, high signal-to-noise ratio, and sharp edge definition.^2^ Despite its high-resolution, it has low acquisition times making it suitable for addition to current protocols without significantly extending the time required for the study. It has been used previously for preoperative planning to better evaluate small structures in abdominal surgery during gestation, in body oncologic imaging, and in craniofacial surgery.^2^^,^^5^^,^^8^^,^^18^ Despite this, FIESTA MRI has been studied sparingly in orthopedic applications limited to the evaluation of lumbar foraminal stenosis with no reported use in the shoulder.^13^

Therefore, the purpose of this study was to determine the correlation of a 2D FIESTA MRI protocol with 3D CT for calculation of GBL. A secondary objective was to determine if a 2D FIESTA protocol had a higher correlation than the commonly used 2D proton density fat-saturation (PD FS) MRI sequence and if there are differences in agreement between imaging modalities across the spectrum of GBL. Our null hypothesis was that 2D FIESTA MRI would have a strong correlation (>0.7) with 3D CT for measurement of GBL, would outperform 2D PD FS MRI sequences, and would do so across a range of subcritical and critical bone loss.

## Materials and methods

**Figure 1:**
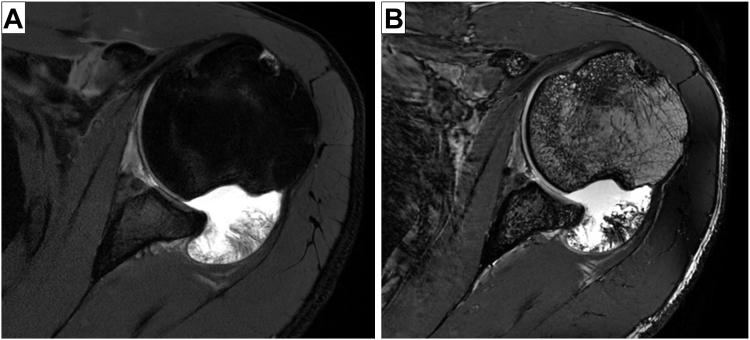
Axial magnetic resonance arthrogram images of a patient with a bony Bankart and large Hill-Sachs lesions on (**A**) proton density fat-saturation and (**B**) fast-imaging employing steady-state acquisition sequences.

Institutional review board approval (WCG IRB #U21-10-4593) was obtained for this retrospective study at a single institution. We queried for patients who underwent a shoulder stabilization procedure for anterior shoulder instability with Current Procedural Terminology codes 23455 (open Bankart repair), 23462 (coracoid process transfer), and 29806 (arthroscopic capsulorrhaphy) from 2010 to 2023. Patients were included if they underwent imaging of the operative shoulder by both 3D CT reconstruction with humeral head subtraction and magnetic resonance (MR) arthrogram performed on a general electric or Philips 3 Tesla scanner using the standard arthrogram protocol with FIESTA sequence (Table I). Patients were excluded if the MRI study did not contain both PD FS and FIESTA sequences (Fig. 1), for posterior shoulder instability or history of shoulder surgery, if the CT and MRI were obtained greater than 90 days apart, or if there was a documented instability event between imaging studies. The order of obtaining CT vs. MRI was not an exclusion criterion. The indications for obtaining imaging were at the discretion of the treating surgeon. We collected patient demographics to include sex, age, and BMI in addition to preoperative instability events differentiated by number of subluxations and dislocations.

**Table I:** FIESTA pulse sequence for magnetic resonance arthrogram.

Characteristic	Value
Acquisition plane	Axial
TR	8.2
TE	Min Full
FOV (cm^2^)	14
Matrix	320 × 320
FA	30
Slice thickness (mm)	0.80
No slab wrap	1.08
Bandwidth (kHz/pixel)	31.25
NEX	1 @ 50% slice resolution
DL	Off
Intensity correction	SCENIC
Acceleration	1.0

*FIESTA*, fast-imaging employing steady-state acquisition magnetic resonance imaging; *TR*, time repetition; *TE*, echo time; *FOV*, field of view; *FA*, fractional anisotropy; *NEX*, number of excitations; *DL*, deep learning..

### Imaging analysis

A junior and senior rater independently performed measurements of GBL twice separated by 3 weeks for temporal blinding to prior measurements. The senior rater (U.R., rater 1) was a fellowship-trained and board-certified musculoskeletal radiologist and the junior rater (S.T., rater 2) was a medical student trained to perform measurements by the senior author (E.C.).

**Figure 2:**
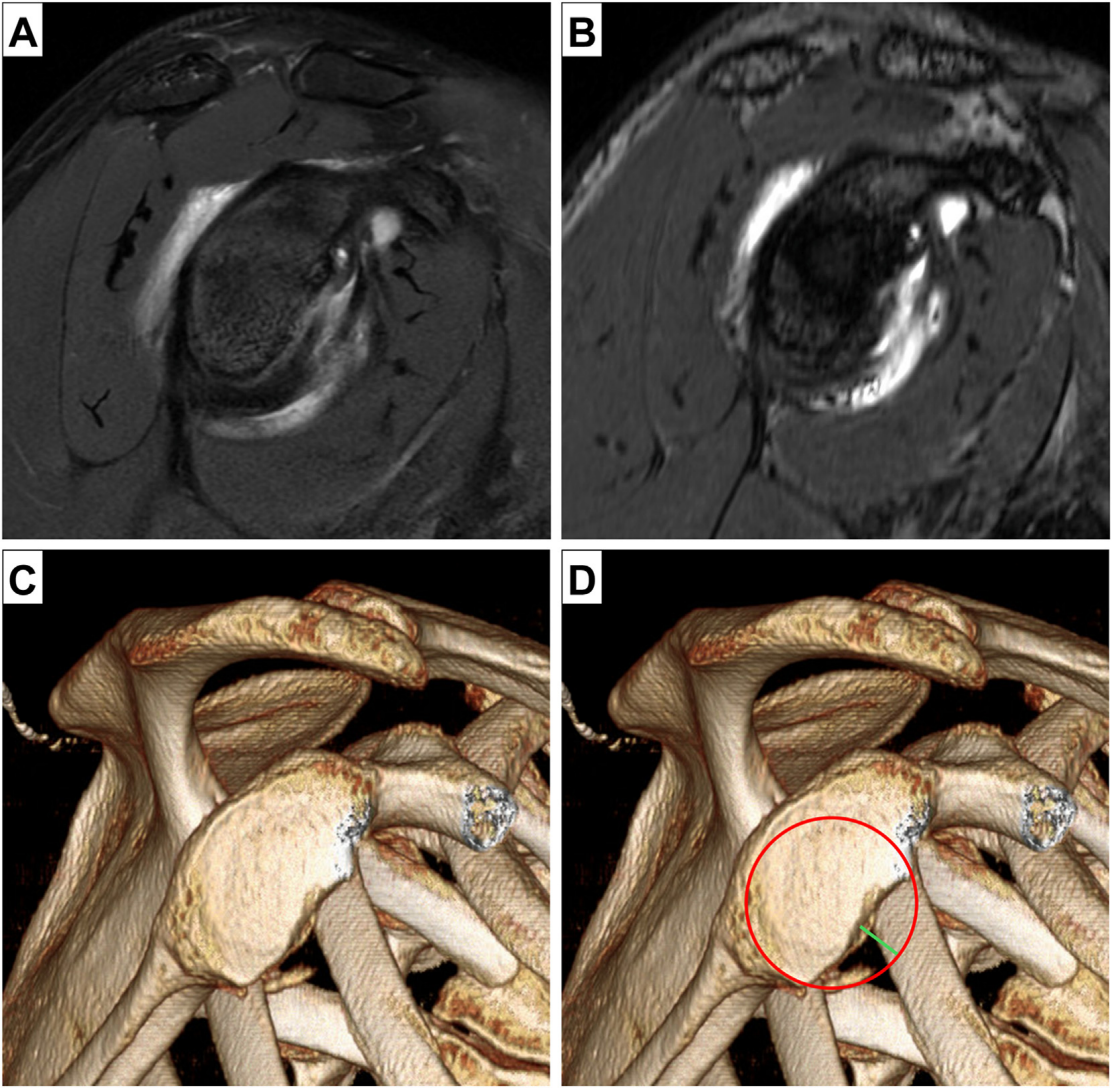
En face sagittal views of a patient with approximately 17% glenoid bone loss after multiplanar reformation on (**A**) PD FS MRI, (**B**) FIESTA MRI, and (**C**) 3D CT reconstruction. (**D**) Best-fit circle linear method for measurement of native glenoid diameter and linear defect to calculate glenoid bone loss (% GBL = defect width/glenoid diameter × 100). The *red circle* matches the contour of the posteroinferior glenoid and the *green line* corresponds to the defect width. This method was performed on all 3 imaging modalities. *MRI*, magnetic resonance imaging; *PD FS*, proton density fat-saturation; *FIESTA*, fast-imaging employing steady-state acquisition; *GBL*, glenoid bone loss; *3D*, three-dimensional; *CT*, computed tomography.

We performed all measurements on Visage (version 7; Visage Imaging, Inc.; Richmond, Melbourne, VIC, Australia) software. The primary outcome was percent GBL measured by the best-fit circle method through overlay of a circle on the inferior glenoid matching the curvature of the posteroinferior glenoid.^9^^,^^24^ The secondary outcome was glenoid diameter and the width of the anteroinferior glenoid defect. GBL was calculated as the ratio of the glenoid defect divided by the best-fit circle diameter. On axial 2D FIESTA and sagittal PD FS MRI series, we utilized the flat multiplanar reformation (MPR) function in Visage to create sagittal realignments for an en face view of the glenoid matching the native glenoid version prior to GBL measurement (Fig. 2).^6^ We defined critical GBL as >15% since it was previously shown that the perfect circle method had a 100% negative predictive value for identifying this threshold of GBL and is within the cited values for critical GBL.^19^

### Sample size estimation

We performed a power analysis for 80% power to detect a strong correlation for the primary outcome between imaging modalities yielding a sample size of n = 11 using the reference study of Vopat et al.^23^ A strong correlation (>0.7) would be necessary to impact clinical practice through substitution of 2D MRI sequences for the current gold standard of 3D CT reconstruction for measurements of GBL.

### Statistical analysis

Descriptive statistics were generated for the radiographic measurements and paired sample t-tests conducted to compare between raters. Shapiro-Wilk normality tests were conducted to evaluate if continuous variables were normally distributed. Intrarater and inter-rater reliability was assessed using intraclass correlation coefficients (ICC) for GBL, linear defect, and glenoid perfect circle diameter. For intrarater reliability, ICC3 and ICC2 were used since the same 2 raters performed all measurements twice on all modalities. For inter-rater reliability, ICC3 models were used since the means of the individual raters' 2 measurements on each modality were compared. We classified ICC values less than 0.5 as poor reliability, values between 0.5 and 0.75 as moderate reliability, values between 0.75 and 0.9 as good reliability, and values greater than 0.90 as excellent reliability. Concordance correlation coefficients (CCCs) were calculated to compare PD FS to 3D CT and FIESTA to 3D CT. Finally, we computed linear regression models to develop equations based on the model β coefficients to predict CT GBL based on PD or FIESTA measured GBL. Once the equations were developed, we input PD and FIESTA GBL measures into the respective equations and then calculated the average percent error between the estimated and actual CT GBL. A-priori alpha level was established as *P* < .05. Statistical analyses were performed using Jamovi (v2.2.5.0; Jamovi Project, Sydney, Australia).

## Results

Twenty-three patients were identified to have 2D PD FS MRI, 2D FIESTA MRI, and 3D CT reconstructions. Eleven were excluded: 7 for >90 days between CT and MRI, 2 for posterior instability, and 2 for prior shoulder surgery. Twelve patients met inclusion and exclusion criteria and were included for analysis. They were 83.3% male with a mean age 31.2 ± 17.2 years and BMI of 24.2 ± 3.7 kg/m^2^. Patients sustained a median zero subluxations (interquartile range [IQR] 2.3), 1 dislocation (IQR 3.0), and 3 instability events (IQR 3.5) prior to obtaining advanced imaging.

Overall mean percent GBL was 12.1 ± 11.2% on PD FS, 11.8 ± 10.0% on FIESTA, and 11.5 ± 9.7% on 3D CT. On 3D CT, the mean percent GBL for each of the 12 patients was 0.0, 0.0, 1.1, 1.6, 3.8, 14.4, 15.0, 16.8, 18.4, 19.5, 20.3, and 27.6%. Descriptive statistics for radiographic measurements by rater and inter-rater and intrarater reliability estimates (ICCs) can be found in Table II. There was at least good inter-rater and intrarater reliability for all measurements and excellent reliability for the primary outcome of % GBL. All CCCs between radiographic measurements on MRI sequences and CT were strong as shown in Table III. The confidence intervals were higher and nonoverlapping for the CCC for % GBL for FIESTA vs. CT compared to PD FS vs CT.

**Table II:** Descriptive statistics for imaging measurements by rater with inter-rater and intrarater reliability.

Imaging measurement	Rater 1 (senior rater): Mean ± standard deviation	Rater 2 (junior rater): Mean ± standard deviation	*P* value	Interrater -ICC3 and 95% CI	Intrarater -ICC3,2 for rater 1 and 95% CI	Intrarater -ICC3,2 for rater 2 and 95% CI
Sagittal PD FS glenoid linear defect [mm]	3.5 ± 3.2	3.4 ± 3.2	.692	0.958 [0.886-0.985]	0.990 [0.971-0.996]	0.994 [0.984-0.998]
Sagittal PD FS glenoid perfect circle diameter [mm]	27.1 ± 2.6	29.2 ± 3.0	.002	0.805 [0.533-0.926]	0.938 [0.826-0.978]	0.979 [0.940-0.992]
Sagittal PD FS % GBL	12.9 ± 11.8	11.4 ± 10.7	.165	0.957 [0.883-0.984]	0.996 [0.988-0.999]	0.996 [0.989-0.999]
Sagittal FIESTA glenoid linear defect [mm]	3.5 ± 3.1	3.3 ± 2.8	.139	0.984 [0.954-0.994]	0.981 [0.945-0.993]	0.993 [0.979-0.997]
Sagittal FIESTA glenoid best-fit circle diameter [mm]	27.1 ± 3.0	28.9 ± 2.9	.005	0.821 [0.566-0.932]	0.921 [0.776-0.972]	0.917 [0.766-0.971]
Sagittal FIESTA % GBL	12.7 ± 11.1	11.0 ± 9.2	.034	0.971 [0.920-0.990]	0.978 [0.937-0.992]	0.994 [0.982-0.998]
CT glenoid linear defect [mm]	3.7 ± 3.2	3.1 ± 2.7	.087	0.937 [0.832-0.977]	0.975 [0.929-0.991]	0.995 [0.987-0.998]
CT glenoid best-fit circle diameter [mm]	27.9 ± 2.6	29.2 ± 3.3	.060	0.754 [0.433-0.905]	0.865 [0.621-0.952]	0.924 [0.785-0.973]
CT % GBL	12.7 ± 10.8	10.4 ± 8.8	.048	0.934 [0.825-0.976]	0.982 [0.950-0.994]	0.997 [0.992-0.999]

*PD FS*, proton density fat-saturation; *GBL*, glenoid bone loss; *CT*, computed tomography; *FIESTA*, fast-imaging employing steady-state acquisition magnetic resonance imaging; *ICC*, intraclass correlation coefficient; *CI*, confidence interval.

**Table III:** Correlation of imaging modalities for imaging measurements.

Imaging measurement	Concordance correlation coefficient (95% CI)
Sagittal PD FS vs. CT	
Glenoid linear defect	0.993 (0.979-0.997)
Glenoid best-fit circle diameter	0.858 (0.588-0.956)
% GBL	0.982 (0.960-0.992)
FIESTA vs. CT	
Glenoid linear defect	0.997 (0.989-0.999)
Glenoid best-fit circle diameter	0.949 (0.837-0.985)
% GBL	0.998 (0.993-0.999)

*PD FS*, proton density fat-saturation; *GBL*, glenoid bone loss; *CT*, computed tomography; *FIESTA*, fast-imaging employing steady-state acquisition magnetic resonance imaging; *CI*, confidence interval.

The mean error for % GBL between PD and CT was −0.592 (95% CI, −1.662, 0.478). The mean error for % GBL between FIESTA and CT was 0.331 (95% CI, −0.315, 0.977). A linear regression model to fit PD FS to CT for calculating GBL indicated that when PD FS measured GBL was compared with CT measured GBL (Equation 1), there was a mean error of 0.005% [95% CI, −0.600, 0.610; R^2^ = 0.988].

Equation 1 CTGBL=(PDGBL×0.860)+1.102

A linear regression model to fit FIESTA to CT for calculating GBL indicated that when FIESTA-measured GBL was compared with CT-measured GBL (Equation 2), there was a mean error of −0.003% [95% CI, −0.625, 0.619; R^2^ = 0.987].

Equation 2 CTGBL=(FIESTAGBL×0.969)+0.682

The largest differences in GBL compared to CT calculated by the regression models were 2.3% for PD FS and 2.5% for FIESTA.

## Discussion

In this comparison of 2D MR arthrogram sequences for calculating GBL against the gold standard of 3D CT, both PD FS and FIESTA sequences were strongly correlated with CT for calculation of GBL across a spectrum of GBL. FIESTA sequences correlated more strongly to CT than PD FS sequences for percent GBL, although both had excellent inter-rater and intrarater reliability with strong concordance. The concordance remained strong between modalities for measuring native glenoid diameter using the best-fit circle method and although the inter-rater and intrarater reliability was less than the reliability for the measurement of the glenoid linear defect, this did not appear to affect the calculation of GBL.

Linear regression models using GBL measured from PD FS and FIESTA to predict GBL on CT were accurate to within 0.6% of actual CT-calculated GBL 95% of the time. These values support the ability of 2D MR arthrogram with PD FS or FIESTA sequences to independently calculate GBL without obtaining an additional CT study. This is in contrast to prior studies which provide evidence for 3D CT as the gold standard to assess GBL.^3^^,^^15^ These studies had the added benefit of a reference standard in caliper measurement of cadaveric glenoid diameter and defects prior to imaging. However, they used 1.5-T noncontrast scans and made no mention of utilizing MPR for obtaining a better en face view. These differences may account for the lower performance of MRI in their studies.

The use of MPR has been shown to improve the reliability of GBL measurements, especially with MRI given its decreased spatial resolution compared to CT.^20^ Changes in version or imaging out of plane to the glenoid articular surface can create error in measurements of GBL. Conventional flat MPR utilized in the current study is widely accessible, although there are also other advances in curved MPR which may further improve upon flat MPR assessment of GBL.^6^ Flat MPR reformats an axial image into a sagittal oblique reconstruction aligned with the anterior and posterior aspects of the glenoid articular surface. Curved MPR, on the other hand, creates a curved sagittal reconstruction along the concavity of the glenoid surface, which has been shown to be more reliable and accurate for glenoid area measurements.^6^

Historical investigations into methods of evaluating GBL pre- and postoperatively are abundant. Despite consensus that 3D CT is the gold standard for evaluation of GBL, it is not without error. This modality along with various measurement methods have been shown to have issues with the accuracy, reproducibility, and heterogeneity of measuring GBL.^19^^,^^22^ This was reflected in the lower reliability for best-fit circle diameter measurements in the current study. However, a recent study demonstrated that GBL may be more reliably measured with a best-fit circle that is 2/3 of the glenoid height measured from the infraglenoid to supraglenoid tubercles.^11^ This differed from the methodology in the current study for creation of the best-fit circle matching the posteroinferior aspect of the glenoid. Our findings of lower reliability between raters and correlation between modalities for the glenoid diameter are similar to their results, indicating that use of a 2/3 glenoid height circle method may further improve reliability over the results reported in the current study and allow for wider adoption for clinicians with image viewing software that need only possess a linear measuring tool.

Recent trends in preoperative utilization of CT and MRI increase cost and burden to the health-care system and patient.^16^ While only 2D MRI was utilized in this study, 3D MRI has also been shown as a viable substitution for 3D CT.^10^^,^^12^^,^^23^^,^^25^ However, the technology used for 3D MRI is not widely available, can require additional resources to post-process, and add cost to the health-care system.^10^^,^^20^ Lander et al evaluated the financial impact at a single institution of using 3D MRI to replace 3D CT for preoperative evaluation of bipolar lesions and found that imaging cost would decrease by $2,670 for 3D MRI, which was 1.67 times cheaper.^10^ This would require MRI acquisition prior to CT and the cost of adding 3D reconstruction was $900 in their study. Use of 2D MRI without a 3D reconstruction for evaluation of GBL may further reduce cost. Although a cost comparison was not performed as part of the present study, the addition of a FIESTA sequence at our institution does not increase cost of the MRI.

### Limitations

The small sample size was powered to show a strong correlation between modalities, although we were limited from performing meaningful subgroup analyses for the novel sequence of FIESTA in evaluation of GBL. The inclusion of several patients with minimal to no bone loss could have also influenced our correlations between modalities. Further investigation with larger cohorts is warranted to determine potential benefits of FIESTA over other MRI sequences. Specifically, cohorts with GBL between 10 and 20% warrant additional investigation to validate our results for operative decision-making. MR arthrogram with MPR was utilized and our results need further validation on 2D noncontrast MRI without MPR. In addition, the en face view of the glenoid on FIESTA sequences was obtained from MPR reformation of an axial sequence, which did visibly degrade the resolution from the original axial sequence in comparison to PD FS reformations which were obtained from sagittal sequences. Further study is warranted for sagittal FIESTA sequences to determine if further improvements are feasible. However, FIESTA was determined to be a viable option in this study despite this and there may be room for further improvement with reformatting of a dedicated sagittal sequence. We did not evaluate intraoperative description or compare arthroscopic visualization of GBL with imaging as a reference for the accuracy of 3D CT as our reference standard. Further, our method of GBL measurement with the ipsilateral perfect circle method may not be interchangeable for other linear, area, or volume measurement techniques for GBL. Lastly, we did not assess glenoid version or bipolar lesions, which may impact utilization of 2D FIESTA MRI exclusively for preoperative planning in cases of posterior shoulder instability or Hill-Sachs lesions warranting graft augmentation.

## Conclusion

2D FIESTA MR arthrogram had strong concordance with 3D CT reconstruction for calculation of GBL and slightly outperformed PD FS sequences, although both had excellent reliability and correlation to 3D CT. Both PD FS and FIESTA sequences are suitable alternatives for calculation of GBL across the spectrum of GBL, although FIESTA likely does not warrant an additional sequence in MRI protocols for the evaluation of GBL when PD FS is already obtained.

## Disclaimers

Funding: No funding was disclosed by the authors.

Conflicts of interest: The authors, their immediate families, and any research foundation with which they are affiliated have not received any financial payments or other benefits from any commercial entity related to the subject of this article.
